# Inflammatory and Immunological parameters in adults with Down syndrome

**DOI:** 10.1186/1742-4933-8-4

**Published:** 2011-04-16

**Authors:** Maria BF Trotta, João B Serro Azul, Mauricio Wajngarten, Simone G Fonseca, Anna C Goldberg, Jorge E Kalil

**Affiliations:** 1 Heart Institute University of São Paulo Medical School, São Paulo; 2 Albert Einstein Hospital, São Paulo

## Abstract

**Background:**

The increase in life expectancy within the general population has resulted in an increasing number of elderly adults, including patients with Down syndrome (DS), with a current life expectancy of about 50 years. We evaluate the parameters of humoral and cellular immune response, the quantitative expression of the regulator of calcineurin1 gene (RCAN1) and the production of cytokines. The study group consisted of adults DS (n = 24) and a control group with intellectual disability without Down syndrome (ID) (n = 21) and living in a similar environmental background. It was evaluated serology, immunophenotyping, the quantitative gene expression of RCAN1 and the production of cytokines.

**Results:**

In the DS group, the results showed an increase in NK cells, CD8, decreased CD19 (p < 0.05) and an increase spontaneous production of IFNgamma, TNFalpha and IL-10 (p < 0.05). There was not any difference in RCAN1 gene expression between the groups.

**Conclusions:**

These data suggest a similar humoral response in the two groups. The immunophenotyping suggests sign of premature aging of the immune system and the cytokine production show a proinflammatory profile.

## Introduction

The increase in life expectancy of the general population has resulted in an increasing number of elderly adults, including patients with Down syndrome (DS), with a current life expectancy of about 50 years [1]. The death causes in adults with DS are different from the ones of the general population. Alzheimer's disease (AD), congenital heart defects, aspiration, pneumonia are among the most frequent when compared to solid tumors and ischemic heart disease [2-6]. The association with leukemia decreases with age and it is not apparent after the age of 40 [1]. Older DS have an increased susceptibility to infections. However, most individuals with this syndrome do not show clear features of immunological diseases. Many of these immunological alterations are age-related changes and can be enclosed in the spectrum of multiple signs of early senescence, which is characteristic of the DS [7]. The immunological response varies in the aging process. In children over the age of 6, the absolute number of IgG and IgA increases. IgM rates decrease in adolescence and throughout the aging process, there is a low number of circulating B cells (CD19), a decrease of CD4+, an increase of CD8 and NK cells. In DS, granulocyte apoptosis is accelerated in various conditions. T-cells with the early apoptotic phenotype were increased in cell cultures from DS children [8,9]. The response against vaccinal antigens and other antigens such as pertussis, rubella, measles, hepatitis A and B was better in DS patients when compared with other groups [10-14]. However other studies disagree with this affirmation [15-18].

It was proposed a model suggesting that, in Down syndrome, the overexpression of chromosome 21 encoded gene products leads to an impairment of the immunological response [19]. However, there are few studies about immunological parameters in older adults with DS. This extra copy of HSA21 may be responsible for the increased expression of many genes encoded on this chromosome. The trisomy of chromosome 21 is characterized by multiple signs of early senescence which justify its inclusion within the group of "segmental progeroid syndromes," defined as those genetic disorders in which multiple major aspects of the senescent phenotype appear [20,21]. The regulator of calcineurin 1 gene, RCAN1, is present in the specific region in the HSA21 that was described as containing the genes responsible for phenotypic characteristics of the syndrome. The RCAN1 gene product interacts physically with calcineurin A, a catalytic subunit of the Ca (2+)/calmodulin-dependent protein phosphatase PP2B and inhibits its activity [22]. The dephosphorylation of the nuclear factors of activated T cells (NFATs) by calcineurin is essential for activating cytokine gene expression and, consequently, the immune response. Although the effects of RCAN1 on the immune system have not yet been directly examined, the therapeutic benefits of other calcineurin inhibitors have been examined in a variety of conditions [23]. Current immunosuppressive protocols are based mainly on the administration of the calcineurin enzyme inhibitors cyclosporine A and FK506 [24]. The RCAN1 gene consists of seven exons, four of which (exons 1-4) can be alternatively spliced to produce a number of different mRNA isoforms, since exons 5-7 are likely to be common in each mRNA isoform. These isoforms may have different expression patterns, functions and regulation mechanisms. The expression of exon 2 was detected in fetal, but not adult, human brain and calcineurin can induce the expression of RCAN1 isoform 4 mRNA, but it does not induce the other isoforms [25]. There is no study examining the quantitative expression of RCAN1 in adults with DS and its association with cytokines production. The purpose of this study was to evaluate the aspects of humoral and cellular responses, as well as, to look into the quantitative expression of the calcineurin1 (RCAN1) regulator with relation to cytokine production in peripheral mononuclear cells in adults with DS and the ones with intellectual disabilities (ID) due to other causes and who were monitored in the same institution, Associação de Pais e Amigos dos Excepcionais (APAE-SP).

## Results

### 1) Serology

The IgG, IgA, IgM, cRP, ASO frequencies and the complement fraction (C3, C4) were similar in the serum of both groups analyzed. Similarly, there were no difference in the antibody levels against cytomegalovirus, mononucleosis, toxoplasmosis and hepatitis B antigens, measles and rubella vaccines (p > 0.05) (Table 1).

**Table 1:** Serology in Down syndrome and intellectual disabilities

Serology	Down syndrome(DS) (%)	Intellectual disabilities (ID) (%)	*p *value*
IgG (mg/dl)	25	5	0.13
IgM, mg/dl	9	30	0.37
IgA, mg/dl	41	15	0.11
C3, mg/dl	35	50	0.49
C4, mg/dl	28	11	0.36
cRP, mg/l	21	52	0.06
ASO, UI/ml	64	33	0.07
Infectious monucleosis (IgG), UA/ml	78	90	0.37
Toxoplasmosis(IgG), UI/ml	35	60	0.29
HepatitisB (AntiHBS)UI/ml	0	4	1
Measles (IgG), UI/ml	100	100	1
Rubella (IgG), UI/ml	71	89	0.36
Cytomegalovirus(IgG), UI/ml	78	79	1

* Fisher test

### 2) Immunophenotype

The CD3 and CD14 percentages were similar in both groups. The results showed lower percentage of B lymphocytes (CD19+) in DS compared to ID individuals. In contrast, higher percentages of CD8+ T cells; ratio CD4:CD8 was (1:1) and NK cells were detected on DS compared to ID individuals with significance difference (p < 0.05); (Table 2).

**Table 2:** Phenotype of peripheral blood mononuclear cells in Down syndrome and Intellectual disabilities individuals

Imunophenotype	Down syndrome(DS) n = 19	Intellectual disabilities(ID) n = 20	p value*
CD3	69.77 ± 8.01	64.86 ± 10.53	0.11
CD4	40.96 ± 12.68	43.48 ± 11.00	0.51
CD8	34.38 ± 15.38*	22.47 ± 8.78*	0.005
CD19	3.63 ± 2.54*	15.28 ± 7.86*	0.001
CD16^+^/CD56^+^/CD3^-^	44.75 ± 23.01*	29.19 ± 15.41*	0.017
CD14	77.88 ± 11.65	84.15 ± 9.987	0.078

*t test

### 3) Cytokine production

**Figure 1:**
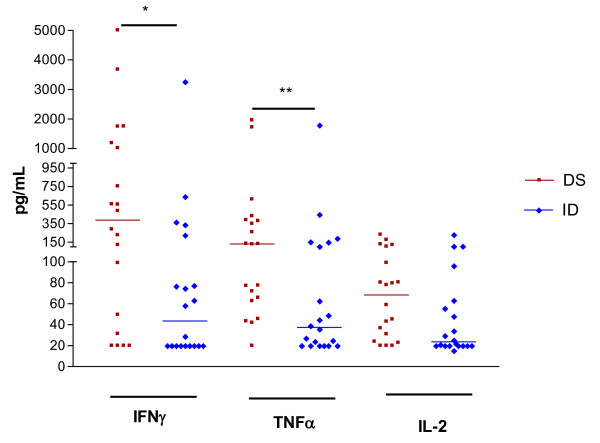
**Spontaneous production of IFNγ, TNFα and IL2 in Down syndrome (DS) and Intellectual disabilities group (ID)**. Periferic blood mononuclear cells were cultured for 48 h in supplemented medium. IFNγ, TNFα and IL2 were detected in the supernatants by CBA. The results are expressed in pg/ml. *p < 0.05. **p < 0.01.

**Figure 2:**
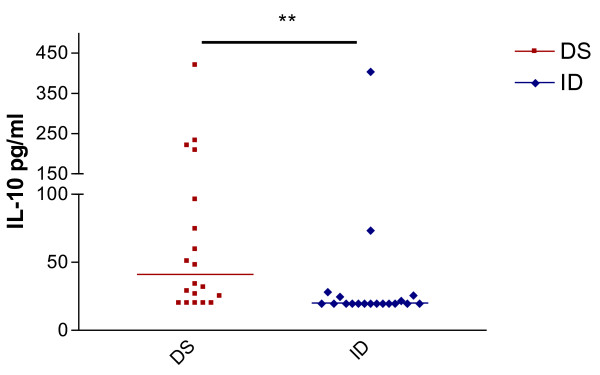
**Spontaneous production of IL-10 in Down syndrome (DS) and Intellectual disabilities group (ID)**. Periferic blood mononuclear cells were cultured for 48 h in supplemented medium. IL-10 was detected in the supernatants by CBA methods. The results are expressed in pg/ml. ** p < 0.01.

The levels of IFNγ, TNFα, IL-10 in the Down syndrome group (DS) were higher than in the intellectual Disabilities (ID) group (p < 0.05). The median value of IFNγ was 389.2 pg/ml in the Down syndrome group (DS) and 43.55 pg/ml in the Intellectual disabilities group (ID) with p = 0.011. The TNFα had medians (DS and ID) of 131.2 pg/ml and 37.38 pg/ml, respectively, with p = 0.0071 and IL-2 had medians of 68.40 pg/ml in DS and 23.55 pg/ml in ID, with p = *ns *(Figure 1). The median value of spontaneous IL-10 production was significantly higher in the DS group (41.00 pg/ml) when compared to the median value (11.10 pg/ml) in ID group with p = 0.006; (Figure 2). Most samples from both groups (DS and ID) had spontaneous production of IL-4 and IL-5 with values below level of detection (< 20 pg/mL).

### 4) Relative Quantification of RCAN1 in PBMC

**Figure 3:**
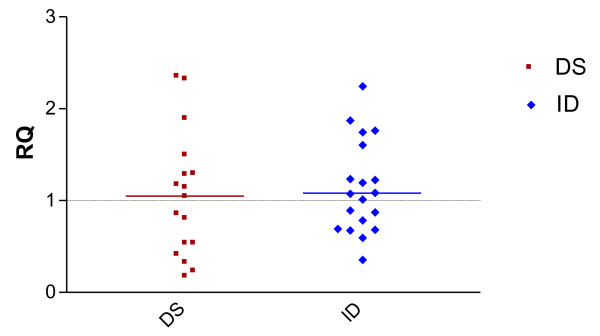
**Relative quantification of RCAN1 in Down syndrome (DS) and Intellectual disability group (ID)**. The median in two groups (DS and ID) were respectively (1.05; 1.08) with p = *ns*.

We studied the expression of RCAN1 in PBMC. The data demonstrate neither negative regulation nor positive regulation of the gene RCAN1; with the medians of (DS and ID) 1.05 and 1.08, respectively with *p = ns*; (Figure 3).

## Discussion

In this study the humoral and cellular aspects of immune response were evaluated, as well as the quantitative expression of the RCAN1 gene in a sample population of adults with DS and in an ID group without Down syndrome with a similar environmental condition. We sought to minimize the external and environmental factors influences.

The results show the humoral and cellular immune response aspects in adults with DS and with ID who were followed at APAE-SP. Since all individuals in this study live in the same community the immunological response differences are not due to the environmental condition.

In this study, the humoral immune response of IgG, IgM, IgA immunoglobulin isotypes, complement factor, cRP, ASO, hepatitis B, cytomegalovirus, measles, rubella, toxoplasmosis, mononucleosis were similar in both groups. Some of the results are in line with the ones of other authors [26,27,17,18] and they may reflect an adaptive mechanism in adults with Down syndrome in order to sustain an efficient response

The immunophenotypic findings in the DS group show increased numbers of NK cells and CD8+ T lymphocyte subpopulation with a ratio of 1:1 CD4:CD8, and decreased numbers of the B lymphocyte (CD19) percentages suggesting a predominance of cells of the innate immune response in DS [28]. Dietary antigens may represent a continuous stimulus for the immune system in this syndrome and interfere with normal immune responses [29].

This data also suggest early immunosenescence and accelerated apoptosis, preserving some subpopulations [9]. Many of these alterations are similar to those characteristic of chromosomally normal subjects of advanced age [30].

The fetal thymic development in the trisomy 16 (Ts16) mouse was examined, which is considered to be a model for human trisomy 21. The Ts16 thymus contains 10 to 20% of the number of lymphocytes found in a normal thymus at a comparable stage [31]. DS thymocytes have a markedly diminished proportion of cells expressing high levels of alpha, beta T cell receptor (alpha, beta TCR) and the associated CD3 molecule. In addition, DS subjects had a lower proportion of CD4 + CD45RA + cells representing naive T cells which have recently emigrated from the thymus [32].

Clinical relevance of this finding is not well established, considering that this immunophenotype is similar to what is found in 26 centenarians [30].

The major age-related modifications were the following: decreased absolute numbers of T lymphocytes (CD3+), involving both CD4+ and CD8+ subsets, accompanied by marked concomitant increased numbers of activated T cells (CD3+, HLA-DR+); marked decreased numbers of B lymphocytes (CD19+) with increase in some subclasses of immunoglobulin and an increase in the number of cells with markers of NK activity [9,33].

Human immunosenescence represents a complex remodeling of the immunological system and it is induced by exposure to antigen and oxidative stress. There was a deterioration of the clonotypical immunity, while ancestral, innate immunity was largely preserved. In an aging immune system, adaptive immunity deteriorates because of a progressive decline of naive T and B cells and also due to a decrease of absolute numbers of T and B lymphocytes. The innate compartment of the immune system is relatively well preserved although some age-dependent alterations can be also observed [30]. Aging is characterized by a peculiar chronic inflammatory status also called 'inflammaging', resulting in cellular and molecular activation, that allows the organism to respond to multiple stress present during the process of aging [30,34].

These data suggest that there is an early immunosenescence probably due to accelerated apoptosis, preserving some subpopulations [9,34,35].

Studies of the T-cell phenotype and function have frequently resulted in conflicting outcomes, the overall evidence strongly pointed to a primary and profound impairment of T-cell mediated immunity [36].

Moreover, the relative and the absolute size of apoptotic CD3+ T lymphocytes, and the relative size of apoptotic CD19+ B lymphocytes were significantly higher in DS children than in controls [9,36].

Several lines of evidence suggest that telomere shortening plays a causal role in cellular aging. If this were true and if telomere shortening plays a role in cellular aging, then one might predict critically shortened telomeres in the lymphocytes of these individuals. It was found that, in DS, the rate of telomere loss in peripheral blood lymphocytes was three times higher than that in age-matched normal donors [37].

In this study the normal immunoglobulin level outcomes, in spite of decreased CD19, indicate an adaptative mechanism.

There are a very few studies about cytokines in DS. Studies in children give evidence that the DS thymus has lower number of phenotypically mature thymocytes, which express high levels of the T cell receptor and associated CD3-molecule [38].

Furthermore, there were high levels of tumor necrosis factor (TNFα) and interferon (IFNγ) cytokines that suggest a deregulation in cytokine production in DS and it may also provide an explanation for the abnormal thymic anatomy and the thymocyte maturation [32]. In adults, high percentages of IFNγ (Th1) were found, and the Th1/Th2 ratio was higher in DS patients than in controls [39]. The elevated production of IFNγ, TNFα and IL-10 was not followed by IL-2 production that has Th1 profile [40]. These cytokines' production can be likely due to the macrophage and to the NK cells and are not produced by T cells.

The plasmatic high levels of pro-inflamatory cytokines in DS patients are due to endothelial activation without the activation of the immune responses [41,42]

There are a very few studies focused in cytokines production in these populations, and none of them link the production of cytokines to RCAN1's overexpression in peripheral blood mononuclear cells.

The overexpression of the RCAN1 gene has already been observed in several tissues, particularly in the human brain, the spinal cord, the kidney, the liver, the mammary glands, the placenta, the skeletal muscles and the heart. In adults, RCAN1 isoform 1 is most highly expressed in the heart, the brain, the muscles and the pancreas, while isoform 4 is most highly expressed in the heart, the liver, the muscles, the placenta, the pancreas and the kidney. The expression of isoform 2 was detected in fetuses [22,43,44]. The RCAN1 is involved in the regulation of various cellular functions [23]. It is overexpressed in the brains of DS fetuses and in post-mortem brain samples of AD patients [20,44]. RCAN1 has also been implicated in cardiac valve formation and in inhibition of cardiac hypertrophy [25]. RCAN1, induced by VEGF, TNF-α, and calcium ionophore, participates in the endothelial cell migration and in the angiogenesis, showing the existence of several regulatory mechanisms [45,46,28].

In this study we used a fragment that includes exon 5 to exon 7 which are present in all isoforms. There are two hypotheses to relate phenotype to gene expression in chromosome 21. Theoretically, the supernumerary copy of human chromosome 21 (HSA21) is expected to result in an increase in the level of transcripts of all genes mapping to HSA21. However, it has been recently observed that there is not always a direct correlation between genomic imbalance (deletion or duplication) and transcript level of genes within the aneuploid segment, suggesting that complex molecular mechanisms regulate RNA transcript levels [47,48]. Due to this reason, many studies have been trying to clear the mechanism that leads to the expression of these genes [49]. A study established that genes in chromosome 21, expressed 1.5 times, would be superexpressed. In respect to gene RCAN1, this finding concludes that an expression under 1.5 indicates that the gene has compensatory mechanisms; in other words, it is influenced by or interacts with other genes [50]. The findings in this study, show that the RCAN1's relative expression is about 1, suggesting that the gene does not interfere in the DS phenotype, which was studied in peripheral blood mononuclear cells. Relative expression of RCAN1 showed neither positive nor negative regulation on the calcineurin pathway and on the production of cytokines.

Maybe other ethics models of study would be necessary with specific stimulus or which would be synergic with other genes to demonstrate the overexpression of RCAN1 in vivo.

## Conclusion

These data suggest a similar humoral response in both groups. In adults with Down's syndrome, the immunophenotypic alterations could be attributed the signals of precocious immunosenescence in the immune system. There is predominance of the Th1 profile in cytokines' production without any apparent relation with the RCAN1's expression in the PBMC. The overexpression of RCAN1 in PBMC was not observed. This study stimulates the evaluation of new ways of studying these genes according to the types of specific stimuli as well as new ways of assessing the influence or interaction with other genes.

## Materials and methods

### Population

The studied population included adults with Down syndrome (DS) or with intellectual disability but without Down syndrome (ID), who were being followed at the same Institution (Associação de Pais e Amigos dos Excepcionais - APAE-SP), and who were living in a similar environmental condition. The blood samples were obtained from 24 patients (DS) with karyotype free of trisomy and 21 patients (ID) without Down syndrome confirmed by karyotype. Mean ages were 38 ± 8,7 years for DS and 43 ± 8,3 years for ID. The collection procedures were approved by the Heart Institute Ethics Committee (Heart Institute, Clinical Hospital, University of São Paulo (HC-FMUSP). The selected patients and/or those responsible for them were informed, in a clear and detailed way, about the objectives of the research and the procedures to be carried out, as well as about the risks, discomforts and benefits, and they signed the Terms of a free, prior and informed Consent.

### Serology

The antibodies to hepatitis B (by MEIA), cytomegalovirus, infectious mononucleosis, toxoplasmosis, rubella (by ELISA), measles (by IFI), and anti-streptolysin O (ASO) were all investigated. the C-reactive protein (cRP), the Complement factors C3, C4, (by nephelometry) and immunoglobulin isotypes IgG, IgM, IgA (by immunoturbidimetric assay) present in the serum from the individuals of the study were also investigated. All serology was performed by the Central Laboratory of Heart Institute, Clinical Hospital, University of São Paulo.

### Cellular Separation and Cell freezing

The venous blood was collected into heparinized tubes (20 mL) and the separation of the peripheral blood mononuclear cells (PBMC) was obtained with Ficoll-Hypaque gradient [51]. A total of 4-5 × 10^6^cells/ml were frozen in appropriate volume of freezing media (10% dimethyl sulphoxide - DMSO, and 90% fetal calf serum, FCS) for posterior flow cytometry analysis.

### Cellular Culture

Peripheral blood mononuclear cells (5 × 10^6 ^cells) were cultured in DMEM (Sigma) containing 20% of fetal calf serum (FCS). After 48 hours the culture supernatants were collected to measure the cytokine production: IL-2, IL-4, IL-5, IL-10, TNFα and IFNγ.

### Cytokine production

Cytokines in culture supernatants were quantified by immunofluorescent flow cytometry (FASCalibur flow cytometer, Becton Dickinson, California, USA) using Human Chemokine and Th1/Th2 Cytokine Cytometric Bead Array (CBA) reagent kits (BD Biosciences Pharmingen, California, USA) according to the manufacturer's instructions. The production of IL-2, IL-4, IL-5, IL-10, TNFα and IFNγ cytokines was quantified. The results were based on a standard concentration curve established between 5000 and 20 pg/ml.

### Flow cytometry analysis

The PBMC cells were thawed and washed for posterior Flow cytometry analysis. The purified PBMC (4-6 × 10^6^cells) were incubated with a panel of fluorochrome-conjugated mAbs. Cells were analyzed using anti-CD8 and andi-CD16-fluorescein isothiocyanate (FITC) (BD Biosciences Pharmingen) anti-CD4, anti-CD19, anti-CD14 and anti-CD56-phycoerythrin (PE) (BD Pharmingen), anti-CD3-indotricarbocyanine coupled with phycoerythrin (PE-Cy5) (BD Pharmingen) monoclonal antibodies. Events (10 × 10^3^) gated were analyzed using a FACScalibur flow cytometer and CellQuest software (Becton-Dickinson, Mountain View, CA). The population region (lymphocyte and monocyte gate) was set manually, based on the forward-scatter and side-scatter characteristics. The relative count of each subpopulation was expressed as a percentage within the total population.

### Quantitative Real-Time PCR (QT-PCR) for RCAN1 gene expression

Total RNA from PBMC was extracted using the Trizol^® ^method (Life Technologies Inc., Grand Island, NY). RNA quantities were determined by A260 measurement and the integrity of the RNA was confirmed by gel electrophoresis. One microgram of total RNA was used for the cDNA synthesis. Briefly, 1 μg of total RNA total, 1 μl oligodT (500 μg/ml), 1 μl dNTP (10 mM of dATP, dCTP, dGTP and dTTP), DEPC water q.s.p. 12 μl, the reaction was conducted for 5 min at 40°C. After this 4 μl Transcription Buffer 5x was added (Tris-HCl 250 mM pH 8,3, KCl 375 mM, MgCl2 15 mM), 2 μl DTT 0,1 M, 1 μl inhibitor RNase (40 U/μl) (RNAse OUT™, Invitrogen, Carlsbad, CA, EUA) and 1 μl Super-script II™ Reverse Transcriptase (200 U/μL) (Invitrogen, Carlsbad, CA, EUA), the reaction was heated for 50 min at 42°C and 15 min at 70°C.

The sequences of primers for QT-PCR were designed using Primer Express (PE Applied Biosystems, Foster City, CA, USA). The following primer sequences were used: RCAN1 (Acc. no. NM_203417) sense 5'-CAGTTTCTGATCTCCCCTCC-3', anti-sense 5'-TCATACTTTTCCCCTGGCCC-3' and Glyceraldehyde-3-phosphate dehidrogenase (GAPDH; Acc. no. M33197) sense 5'-TGGTCTCCTCTGACTTCAACA-3', anti-sense 5'-AGCCAAATTCGTTGTCATACC-3'. The primer sequence design for the RCAN1 gene was performed covering exons 5, 6 and 7 which are constant in all the isoforms of the gene. Real-time PCR reactions were carried out in an ABI Prism 7500 Sequence Detection system (PE Applied Biosystems, Foster City, CA, USA). QT-PCR was performed using the SYBR Green PCR Master Mix (PE Applied Biosystems), according to the manufacturer's instructions. Quantities of specific mRNA in the sample were measured according to the corresponding gene-specific standard curve. All the samples were tested in triplicate with the reference gene GAPDH, a housekeeping gene for normalization of data. For all genes we constructed standard curves and determined the slope to calculate the PCR efficiency according to Pfaffl; (data not show) [52,53]. Normalization and fold change were calculated with the ΔΔCt method with GAPDH as the reference mRNA [54].

#### Statistical Analysis

Statistical analysis was performed by AlphPrism 3.0 and SPSS 16 software. For continuous data with normal distribution, the impaired T test was used. For data which do not include normal distribution, the Mann Whitney test was used. For categorical data, the Fisher test or Chi-square test were used. Statistical significance was given for p < 0.05.

## List of abbreviations

AD: Alzheimer disease; DS: Down syndrome; IgG: ImmunoglobulinG; IgA: Immunoglobulin A; IgM: Immunoglobulin M; ID: Intellectual Disabilities; NK: Natural Killer; PBMC: Peripheral blood mononuclear cells; HSA21: Trisomy of chromosome 21; RCAN1: The regulator of calcineurin 1 gene; NFATs: The nuclear factors of activated T cells; ASO: Streptolysin A; IFNγ: IFNgamma: γ Interferon; TNFα: Tumor necrosis factor α: TNFalpha

## Competing interests

The authors declare that they have no competing interests.

## Authors' contributions

MBFT; ACG: carried out the molecular genetic studies, participated in the sequence alignment and drafted the manuscript.

MBFT; SGF: carried out the immunoassays.

MBFT; ACG: participated in the sequence alignment.

MBFT; JBS: participated in the design of the study and performed the statistical analysis.

JEK; JBSA; MW; ACG conceived of the study, and participated in its design and coordination.

All authors read and approved the final manuscript.

## References

[B1] Yang Q Rasmussen SA Friedman JM Mortality associated with Down's syndrome in the USA from 1983 to 1997: a population-based study Lancet 2002 359 1019 25 10.1016/S0140-6736(02)08092-3 11937181

[B2] Hasle H Clemmensen IH Mikkelsen M Risks of leukaemia and solid tumours in individuals with Down's syndrome Lancet 2000 355 165 69 10.1016/S0140-6736(99)05264-2 10675114

[B3] Licastro F Marocchi A Penco S Porcellini E Lio D Dogliotti G Corsi MM Does Down's syndrome support the homocysteine theory of atherogenesis? Arch Gerontol Geriatr 2006 43 3 381 7 10.1016/j.archger.2006.01.003 16533539

[B4] Ylä-Herttuala S Luoma J Nikkari T Kivimäki T Down's syndrome and atherosclerosis Atherosclerosis 1989 76 2-3 269 72 10.1016/0021-9150(89)90110-X 2525042

[B5] Murdoch JC Rodger JC Rao SS Fletcher CD Dunnigan MG Down's syndrome: an atheroma free-model? British Medical Journal 1977 2 226 228 10.1136/bmj.2.6081.226 141966 PMC1631400

[B6] Dogliotti G Galliera E Licastro F Corsi MM Age-related changes in plasma levels of BDNF in Down syndrome patients Immunity & Ageing 2010 7 2 10.1186/1742-4933-7-2 20181009 PMC2841579

[B7] Cuadrado E Barrena MJ Immune dysfunction in Down syndrome: primary immune deficiency or early senescence of the immune system? Clin Immunol Immunopathol 1996 78 209 14 10.1006/clin.1996.0031 8605695

[B8] Yasui K Shinozaki K Nakazawa T Agematsu K Komiyama A Presenility of granulocytes in Down syndrome individuals Am J Med Genet 1999 84 406 12 10.1002/(SICI)1096-8628(19990611)84:5<406::AID-AJMG4>3.0.CO;2-4 10360394

[B9] Corsi MM Ponti W Venditti A Ferrara F Baldo C Chiappelli M Licastro F Proapoptotic activated T-cells in the blood of children with Down's syndrome: relationship with dietary antigens and intestinal alterations Int J Tissue React 2003 25 3 117 25 14756193

[B10] Li Volti S Mattina T Mauro L Bianca S Anfuso S Ursino A Mollica F Safety and effectiveness of an acellular pertussis vaccine in subjects with Down's syndrome Child Nerv Syst 1996 12 100 2 10.1007/BF00819505 8674075

[B11] McKay E Hems G Massie A Moffat MA Phillips KM Serum antibody to poliovirus in patients in a mental deficiency hospital, with particular reference to Down's syndrome J Hyg (Lond) 1978 81 25 30 10.1017/S0022172400053730 211162 PMC2129762

[B12] Epstein LB Philip R Abnormalities of the immune response to influenza antigen in Down syndrome (trisomy 21) Prog Clin Biol Res 1987 246 163 82 2958879

[B13] Philip R Berger AC McManus NH Warner NH Peacock MA Epstein LB Abnormalities of the *in vitro *cellular and humoral responses to tetanus and influenza antigens with concomitant numerical alterations in lymphocyte subsets in Down syndrome (trisomy 21) J Immunol 1986 136 1661 7 2419411

[B14] Costa-Carvalho BT Martinez RM Dias AT Kubo CA Barros-Nunes P Leiva L Solé D Carneiro-Sampaio MM Naspitz CK Sorensen RU Antibody response to pneumococcal capsular polysaccharide vaccine in Down syndrome patients Braz J Med Biol Res 2006 39 1587 92 10.1590/S0100-879X2006005000047 17160268

[B15] Ferreira CT Leite JC Taniguchi A Vieira SM Pereira-Lima J da Silveira TR Immunogenicity and safety of an inactivated hepatitis A vaccine in children with Down syndrome J Pediatr Gastroenterol Nutr 2004 39 337 40 10.1097/00005176-200410000-00007 15448421

[B16] Hawkes RA Boughton CR Schroeter DR The antibody response of institutionalized Down's syndrome patients to seven microbial antigens Clin Exp Immunol 1978 31 298 304 148342 PMC1541221

[B17] Troisi CL Heiberg DA Hollinger FB Normal immune response to hepatitis B vaccine in patients with Down's syndrome. A basis for immunization guidelines JAMA 1985 254 3196 9 10.1001/jama.254.22.3196 2933533

[B18] do Canto CL Granato CF Garcez E Villas Boas LS Fink MC Estevam MP Pannuti CS Cytomegalovirus infection in children with Down syndrome in a day-care center in Brazil Rev Inst Med Trop Sao Paulo 2000 42 179 83 10.1590/S0036-46652000000400001 10968879

[B19] Murphy M Insoft RM Pike-Nobile L Epstein LB A hypothesis to explain the immune defects in Down syndrome Prog Clin Biol Res 1995 393 147 67 8545448

[B20] Roberts-Thomson IC Whittingham S Youngchaiyud U MacKay IR Ageing, immune response and mortality Lancet 1974 2 368 10.1016/S0140-6736(74)91755-3 4136513

[B21] Martin GM Genetic modulation of senescent phenotypes in Homo sapiens Cell 2005 120 4 523 32 10.1016/j.cell.2005.01.031 15734684

[B22] Fuentes JJ Genesca L Kingsbury TJ Cunningham KW Perez-Riba M Stivill X la Luna S DSCR1 overexpressed in Down syndrome, is an inhibitor of calcineurin-mediated signaling pathways Human Molecular Genetics 2000 11 9 1681 90 10.1093/hmg/9.11.1681 10861295

[B23] Harris CD Ermak G Davies KJ DSCR1 (Adapt78 or RCAN1) in disease Cell Mol Life Sci 2005 62 2477 2486 10.1007/s00018-005-5085-4 16231093 PMC11139107

[B24] Fruman DA Klee CB Bierer BE Burakoff SJ Calcineurin phosphatase activity in T lymphocytes is inhibited by FK 506 and cyclosporin A Proc Natl Acad Sci USA 1992 89 3686 90 10.1073/pnas.89.9.3686 1373887 PMC525555

[B25] Yang J Rothermel B Vega RB Frey N McKinsey TA Olson EN Bassel-Duddy R Williams RS Independent signals control expression of the calcineurin inhibitory proteins MCIP1 and MCIP2 in striated muscles Circ Res 2000 87 E61 68 11110780 10.1161/01.res.87.12.e61

[B26] Burgio GR Ugazio AG Nespoli L Marcioni AF Bottelli AM Pasquali F Derangements of immunoglobulin levels, phytohemagglutinin responsiveness and T and B cell markers in Down's syndrome at different ages Eur J Immunol 1975 5 600 3 10.1002/eji.1830050904 11993318

[B27] Seger R Buchinger G Ströder J On the influence of age on immunity in Down's syndrome Eur J Pediatr 1977 124 77 87 10.1007/BF00477543 137810

[B28] Cossarizza A Monti D Montagnani G Ortolani C Masi M Zannotti M Franceschi C Precocious aging of the immune system in Down syndrome: alterations of B lymphocytes, T-lymphocyte subsets, and cells with natural killer markers Am J Med Genet Suppl 1990 7 213 8 2149950 10.1002/ajmg.1320370743

[B29] Licastro F Mariani RA Faldella G Carpenè E Guidicini G Rangoni A Grilli T Bazzocchi G Immune-endocrine status and coeliac disease in children with Down's syndrome: relationships with zinc and cognitive efficiency Brain Res Bull 2001 55 2 313 7 10.1016/S0361-9230(01)00476-2 11470333

[B30] Franceschi C Bonafè M Centenarians as a model for healthy aging Biochem Soc Trans 2003 31 457 61 10.1042/BST0310457 12653662

[B31] Ewart JL Auerbach R Defects in thymocyte differentiation and thymocyte-stromal interactions in the trisomy 16 mouse Dev Immunol 1992 2 215 26 10.1155/1992/72627 1378332 PMC2275860

[B32] Murphy M Friend DS Pike-Nobile L Epstein LB Tumor necrosis factor alpha and IFN-gamma expression in human thymus J Immunol 1992 149 2506 12 1388194

[B33] Sansoni P Cossarizza A Brianti V Fagnoni F Snelli G Monti D Marcato A Passeri G Ortolani C Forti E Fagiolo U Passeri M Franceschi C Lymphocyte Subsets and Natural Killer Cell Activity in Healthy Old People and Centenarians Blood 1993 82 2767 73 8219229

[B34] Elsayed SM Elsayed GM Phenotype of apoptotic lymphocytes in children with Down syndrome Immunity & Ageing 2009 6 2 10.1186/1742-4933-6-2 19267926 PMC2657904

[B35] Kaszubowska L Telomere shortening and ageing of the immune system J Physiol Pharmacol 2008 59 Suppl 9 169 86 19261979

[B36] Ugazio AG Maccario R Notarangelo LD Burgio GR Immunology of Down syndrome: A review Am J Med Genet 1990 204 12 10.1002/ajmg.1320370742 2149949

[B37] Vaziri H Schächter F Uchida I Wei L Zhu X Effros R Cohen D Harley CB Loss of telomeric DNA during aging of normal and trisomy 21 human lymphocytes Am J Hum Genet 1993 52 661 7 8460632 PMC1682068

[B38] Murphy M Lempert MJ Epstein LB Decreased level of T cell receptor expression by Down syndrome (trisomy 21) thymocytes Am J Med Genet 1990 7 234 7 10.1002/ajmg.1320370747 2149954

[B39] Franciotta D Verri A Zardini E Andreoni L De Amici M Moratti R Nespoli L Interferon-gamma- and interleukin-4-producing T cells in Down's syndrome Neurosci Lett 2006 395 67 70 10.1016/j.neulet.2005.10.048 16289322

[B40] Lio D Candore G Crivello A Scola L Colonna-Romano Cavallone L Hoffmann E Caruso M Licastro F Caldarera CM Branzi A Francheschi C Caruso C Opposite effects of interleukin 10 common gene polymorphisms in cardiovascular diseases ans in sucessful ageing: genetic background of male centenarians is protective against coronary heart disease J Med Genet 2004 41 790 94 10.1136/jmg.2004.019885 15466015 PMC1735604

[B41] Licastro F Chiappelli M Porcellini E Trabucchi M Marocchi A Corsi MM Altered vessel signalling molecules in subjects with Down's syndrome Int J Immunopathol Pharmacol 2006 19 1 181 5 16569356

[B42] Licastro F Chiappelli M Ruscica M Carmelli V Corsi MM Altered cytokine and acute phase response protein levels in the blood of children with Down syndrome Int J Immunopathol Pharmacol 2005 18 165 172 15698521 10.1177/039463200501800117

[B43] Fuentes JJ Pritchard MA Estivill X Genomic organization, alternative splicing and expression patterns of the DSCR1 (Down syndrome candidate region 1) gene Genomics 1997 44 358 361 10.1006/geno.1997.4866 9325060

[B44] Ermak G Morgan TE Davies KJ Chronic overexpression of the calcineurin inhibitory gene DSCR1 (Adapt78) is associated with Alzheimer's disease J Biol Chem 2001 276 38787 94 10.1074/jbc.M102829200 11483593

[B45] Lizuka M Abe M Shiiba K Sasaki I Sato Y Down syndrome candidate region 1, a downstream target of VEGF, participates in endothelial cell migration and angiogenesis J Vasc Res 2004 4 334 44 10.1159/000079832 15263820

[B46] Kim YS Cho KO Lee HJ Kim SY Sato Y Cho YJ Down Syndrome Candidate Region 1 Increases the Stability of the IκBα Protein. *IMPLICATIONS FOR ITS ANTI-INFLAMMATORY EFFECTS** The Journal of Biological Chemistry 2006 281 39051 61 10.1074/jbc.M604659200 17062574

[B47] Saran NG Pletcher MT Natale JE Cheng Y Reeves RH Global disruption of the cerebellar transcriptome in a Down syndrome mouse model Hum Mol Genet 2003 12 2013 19 10.1093/hmg/ddg217 12913072

[B48] Shapiro BL Developmental instability of the cerebellum and its elevance to Down syndrome J Neural Transm 2001 61 Suppl 11 34 10.1007/978-3-7091-6262-0_2 11771737

[B49] Patterson D Molecular genetic analysis of Down syndrome Hum Genet 2009 126 195 214 10.1007/s00439-009-0696-8 19526251

[B50] Aït Yahya-Graison E Aubert J Dauphinot L Rivals I Prieur M Golfier G Rossier J Personnaz L Creau N Bléhaut H Robin S Delabar JM Potier MC Classification of human chromosome 21 gene-expression variations in Down syndrome: impact on disease phenotypes Am J Hum Genet 2007 81 475 91 10.1086/520000 17701894 PMC1950826

[B51] Boyum A Separation of blood leucocytes, granulocytes and lymphocytes Tissue Antigens 1974 4 269 74 10.1111/j.1399-0039.1974.tb00252.x 4415728

[B52] Pfaffl MW A new mathematical model for relative quantification in real-time RT-PCR Nucleic Acids Res 2001 29 e45 10.1093/nar/29.9.e45 11328886 PMC55695

[B53] Pfaffl MW Horgan GW Dempfle L Relative expression software tool (REST) for group-wise comparison and statistical analysis of relative expression results in real-time PCR Nucleic Acids Res 2002 30 e36 10.1093/nar/30.9.e36 11972351 PMC113859

[B54] Livak KJ Schmittgen TD Analysis of relative gene expression data using real-time quantitative PCR and the 2 (-Delta Delta C(T)) Method Methods 2001 25 402 8 10.1006/meth.2001.1262 11846609

